# Laparoscopic versus open radical cystectomy for patients with bladder cancer over 75-year-old: a prospective randomized controlled trial

**DOI:** 10.18632/oncotarget.15717

**Published:** 2017-02-24

**Authors:** Chen Yong, Chen Daihui, Zhou Bo

**Affiliations:** ^1^ Department of Urology, The First Affiliated Hospital of Chongqing Medical University, Chongqing, China; ^2^ Department of Urology, Department of Urology, Daping Hospital, Third Military Medical University, Chongqing, China

**Keywords:** laparoscopic, open radical cystectomy, elderly patients, bladder cancer

## Abstract

The aim of this study was to compare the morbidity, mortality, oncological results and quality of life between laparoscopic radical cystectomy (LRC) and open radical cystectomy (ORC) in the elderly patients over 75 years old. Between January 2012 and January 2015, 60 patients were recruited into this study, who were randomly assigned in a 1:1 ratio to either LRC or ORC group. Baseline patient characteristics, pathological factors, operative and postoperative characteristics, postoperative complications and survival data were retrospectively collected, analyzed and compared between the two groups. Patients in LRC group and ORC group had comparable baseline characteristics and pathological factors (all *P* > 0.05). LRC group required longer operative time (408.2 ± 76.9 vs. 311.7 ± 65.3 min, *P* = 0.000) and had less EBL (621.6 ± 100.7 vs. 1088.5 ± 109.4 ml, *P* = 0.000) compared with ORC group. The incidence of infection and ileus within 90 days after surgery in ORC group was significantly higher than LRC group(6.9% vs. 28.6%, *P* = 0.041; 3.4% vs. 25%, *P* = 0.025). At a median follow-up of 28 months (range 12–48 months), the survival analysis showed that there were no significant differences between the LRC and ORC groups in overall survival (log-rank χ^2^ = 0.122; *P* = 0.726), or progress-free survival (log-rank χ^2^ = 0.153; *P* = 0.696). In conclusion, this study confirmed that LRC could achieve similar tumor treatment efficacy compared to ORC, with fewer perioperative complications and less blood loss. We suggest that LRC should be considered as the primary intervention for patients aged over 75 years old with muscle invasive bladder cancer or non-muscle invasive bladder cancer with high risk factors.

## INTRODUCTION

Bladder cancer (BC), characterized by high risk of recurrence and mortality, is among the fifth most common malignancies in the world [1]. As we all know, there is a strong correlation between the incidence of cancer and age, including bladder cancer. With the gradual aging of the population, the incidence of bladder cancer was rising with the increasing number of older people in China, ranking 1st in the urinary malignant tumor and reaching a peak over age 75 with an incidence of 69.7/100,000 [2]. Therefore, the treatment for the elderly bladder cancer is still an important problem.

Radical cystectomy (RC) combined with pelvic lymphadenectomy is a standard treatment for myometrial invasive bladder cancer, which can improve the survival rate of patients with invasive bladder cancer and avoid local recurrence and distant metastasis [3]. Open radical cystectomy (ORC) has definite curative effect, but there are many deficiencies, such as trauma, blood loss, slow recovery and so on [4]. Elderly patients, especially those over 75 years old, usually have more comorbidities, and therefore have a higher incidence of complications and mortality. As a result, older patients may potentially be guided toward conservative therapies such as radiotherapy, which is not as effective as RC. Therefore, it is necessary to find a more effective and appropriate treatment method for the elderly patients with bladder cancer.

At present, laparoscopic radical cystectomy (LRC) has been widely accepted by most urologists as a minimally invasive treatment, which could reduce the morbidity after conventional surgery [5]. A number of clinical studies have shown that LRC has many advantages, such as a clear vision, precise operation, small trauma and rapid recovery [6–7], since Parra first used laparoscopic surgery for RC in 1992. In addition, many studies have demonstrated the feasibility of laparoscopic radical cystectomy (LRC) was technically feasible and oncological safe [8–9]. However, the elderly patients confront several challenges to LRC surgery such as whether they can tolerate longer operation time, pneumoperitoneum, and peculiar surgical position as well as younger patients [10]. However, there are few studies comparing the feasibility between LRC and ORC for elderly patients over 75 years old [9–11]. To compare the morbidity, mortality, oncological results and quality of life between LRC and ORC in the elderly patients over 75 years old, we conducted a prospective open-label, randomly controlled trial.

## RESULTS

### Baseline patient characteristics

The deadline for follow-up was December 2015. The median follow-up time was 28 months (range 12–48 months). One patient in the LRC group and two patients in the ORC group were lost to follow-up. Therefore, 29 patients in the LRC group and 28 patients in the ORC group were included in the final analysis.

As is shown in Table 1, patients in LRC group and ORC group had comparable baseline characteristics, including age, gender, BMI, Hb, SCR, ALB, Comorbid conditions and ASA class (all *P* > 0.05).

**Table 1:** Baseline patient characteristics

Characteristics	LRC group (*n* = 29)	ORC group (*n* = 28)	χ2/t	*P* value
Age (Median, Range)	78 (75–80)	77 (75–79)	1.003*	0.392
Gender (male/female)	20/9	19/9	0.008	1.000
BMI (kg/m^2^)	22.1 ± 2.8	21.8 ± 3.0	1.293*	0.275
Hb (g/L)	120.8 ± 16.6	123.7 ± 13.5	0.947*	0.496
SCR (umol/L)	103.4 ± 17.3	99.2 ± 12.1	1.287*	0.298
ALB (g/L)	37.1 ± 4.5	36.9 ± 6.1	1.346*	0.204
Comorbid conditions				
Hypertension	7 (24.1%)	10 (35.7%)	0.912	0.395
Diabetes mellitus	9 (31.0%)	6 (21.4%)	0.678	0.550
Coronary heart disease	3 (10.3%)	1 (3.6%)	1.002	0.611
COPD	2 (6.9%)	2 (7.1%)	0.001	1.000
Other chronic diseases	2 (6.9%)	1 (3.6%)	0.316	1.000
ASA class				
2	14 (48.3%)	15 (53.6%)	0.160	0.793
3	15 (51.7%)	13 (46.4%)		

* *t* value.

### Pathological factors

Pathological factors of the two groups are shown in Table 2. Tumor stage, grade, lymph node status, lymph node number and positive surgical margins between the two groups have no significant difference (all *P* > 0.05).

**Table 2:** Pathological factors

Pathological factors	LRC group (*n* = 29)	ORC group (*n* = 28)	χ2/t	*P* value
Tumor stage				
T0, Ta, Tis, T1	11 (37.9%)	9 (32.1%)		
T2	9 (31.0%)	10 (35.7%)	1.135	0.769
T3	4 (13.8%)	6 (21.4%)		
T4	5 (17.2%)	3 (10.7%)		
Grade				
Low	2 (7.1%)	1 (3.6%)	0.316	1.000
High	27 (92.9%)	27 (96.4%)		
Lymph node status				
Negative	24 (82.8%)	22 (78.6%)	0.160	0.747
Positive	5 (17.2%)	6 (21.4%)		
Lymph node number	10.3 ± 2.5	8.6 ± 1.9	0.335	0.954
Positive surgical margins	0 (0.0%)	0 (0.0%)	0.000	1.000

* *t* value.

### Operative and postoperative characteristics

All patients in LRC group were not transferred to open surgery. Operative and postoperative characteristics of the two group are shown in Table 3. Operative time of LRC group was significantly longer than that of ORC group (408.2 ± 76.9 *vs*. 311.7 ± 65.3 min, *P* = 0.000). EBL of LRC group was significantly less than that of ORC group (621.6 ± 100.7 *vs*. 1088.5 ± 109.4 ml, *P* = 0.000). Compared with preoperative data, Hb of LRC group significantly decreased 17.2 ± 3.1 g/L, while that of ORC group also decreased 25.2 ± 4.7 g/L (both *P* < 0.05). ALB of LRC and ORC group both significantly decreased 7.8 ± 1.4 and 9.4 ± 2.1 g/L (both *P* < 0.05). However, there was no significantly change for SCR in both LRC and ORC group. Transfusion needed, postoperative Hb, SCR and ALB, time to liquid intake, time to nasogastric tube removal, time to canalization, time to exsufflation and hospital stay after surgery all have no significant difference between LRC and ORC group.

**Table 3:** Operative and postoperative characteristics

Characteristics	LRC group (*n* = 29)	ORC group (*n* = 28)	χ2/t	*P* value
Operative time (min)	408.2 ± 76.9	311.7 ± 65.3	19.531	0.000
EBL (ml)	621.6 ± 100.7	1088.5 ± 109.4	22.754	0.000
Transfusion needed	7	10	0.160*	0.747
Hb (g/L)	103.6 ± 12.4	98.5 ± 11.7	1.485	0.173
SCR (umol/L)	102.7 ± 35.2	97.9 ± 23.6	0.751	0.519
ALB (g/L)	29.3 ± 3.1	27.5 ± 4.7	0.543	0.694
Time to liquid intake (d)	2.6 ± 0.7	3.1 ± 0.5	1.238	0.301
Time to nasogastric tube removal (d)	1.5 ± 0.3	1.8 ± 0.7	1.527	0.096
Time to canalization (d)	11.2 ± 3.2	11.5 ± 2.7	1.539	0.087
Time to exsufflation (d)	3.6 ± 0.2	4.0 ± 0.5	1.386	0.194
Hospital stay after surgery (d)	14.7 ± 2.3	15.6 ± 3.1	0.874	0.491

* χ^2^ Value.

### Postoperative complications

There were no deaths in the two groups during the perioperative period. Postoperative complications of the two groups were shown in Table 4. Infection is the most common early postoperative complication. The incidence of infection in ORC group was significantly higher than LRC group (6.9% vs. 28.6%, *P* = 0.041). The incidence of ileus within 90 days after surgery was significantly higher in the ORC group than in the LRC group (3.4% vs. 25%, *P* = 0.025). There was no significant difference between the two groups in the incidence of other early complications, including anastomotic leak, delirium, arrhythmia and wound dehiscence. One patient in LRC group suffered from anastomotic leak and was treated by fistula resection and Intestinal anastomosis. Two patients in ORC group suffered from anastomotic leak, of whom one was treated by fistula resection and ileostomy, and the other one with urine leakage from ileo-ureteral anastomosis healed spontaneously. There was no significant difference between the two groups in the incidence of late complications, including pyelonephritis, ileus, ureteral stricture and incisional hernia (all *P* > 0.05). One patient in LRC group and two patients in ORC group required re-operation (3.4%vs. 14.3%, *P* = 0.194).

**Table 4:** Postoperative complications of the two groups

Complications	LRC group (*n* = 29)	ORC group (*n* = 28)	χ^2^	*P* value
Early complications				
Infection	2 (6.9%)	8 (28.6%)	4.626	0.041
Ileus	1 (3.4%)	7 (25.0%)	5.484	0.025
Anastomotic leak	1 (3.4%)	2 (7.1%)	0.390	0.611
Delirium	2 (6.9%)	4 (14.3%)	0.826	0.423
Arrhythmia	3 (10.3%)	2 (7.1%)	0.183	1.000
Wound dehiscence	0 (0.0%)	3 (10.7%)	3.280	0.112
Late complications				
Pyelonephritis	3 (10.3%)	4 (14.3%)	0.205	0.706
Ileus	1 (3.4%)	2 (7.1%)	0.390	0.611
Ureteral stricture	1 (3.4%)	2 (7.1%)	0.390	0.611
Incisional hernia	0 (0.0%)	1 (3.6%)	1.054	0.491
Re-operation required	1 (3.4%)	4 (14.3%)	2.091	0.194

### Survival analysis

**Figure 1:**
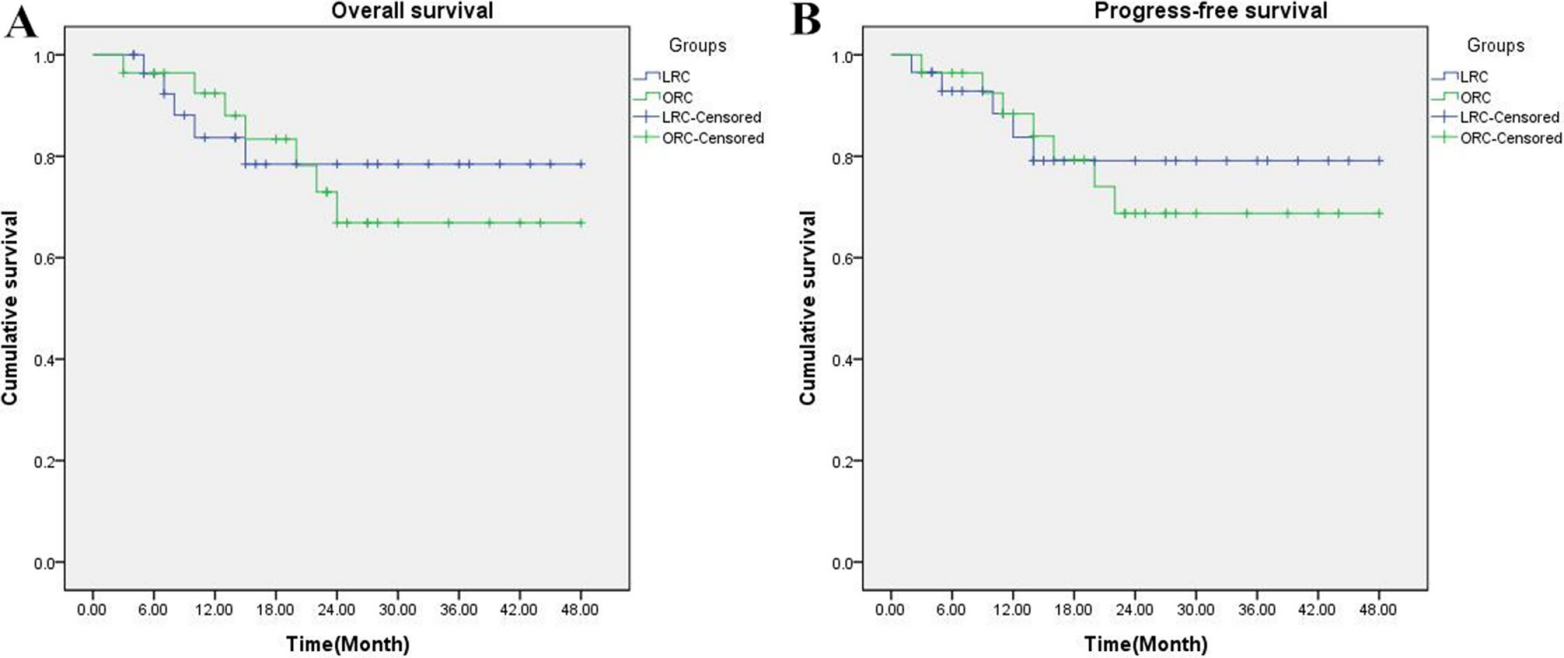
Kaplan-Meier OS (**A**) and PFS survival (**B**) curves for LRC group and ORC group. Both rates were similar for the two groups (log-rank, *P* = 0.726 and *P* = 0.696, respectively).

During the follow-up period, 5 (17.2%) patients in LRC group and 7 (25.0%) patients in ORC group died of bladder cancer. Mortality rates for the two groups did not significantly differ (χ^2^ = 0.516, *P* = 0.530). At the time of this report, all survival in both groups were disease-free. The survival curves of the two groups are shown in Figure 1, which demonstrate that there were no significant differences between the LRC and ORC groups in overall survival (log-rank χ^2^ = 0.122; *P* = 0.726), or progress-free survival (log-rank χ^2^ = 0.153; *P* = 0.696).

## DISCUSSION

The incidence of bladder cancer increased with age increasing [12]. It is reported that the incidence of bladder cancer in people aged ≥ 75 years is 11 times higher than that of those aged < 75 years [12]. With aging of the population, more and more elderly patients with invasive or recurrent bladder cancer need radical surgery. However, the elderly patients with more basic diseases, poor storage of organ physiological function, and decreased immunity caused by surgical damage, would withstand greater risk than young patients for the implementation of radical surgery. Perioperative mortality and complication rates were positively correlated with age. It is reported that the mortality of patients with age 65–69, 70–79 and ≥ 80 years old within 90 days after surgery is 6.1%, 10.1% and 14.8%, respectively [13]. The incidence of postoperative complications was 1.66 times higher in patients aged ≥ 75 years than patients < 75 years old. [14] Therefore, clinical decision-making for elderly patients is a very important and difficult challenge.

Minimally invasive advantage of LRC has been widely recognized under the premise of complying with principle of traditional radical surgery. LRC has the advantages of small trauma and rapid recovery, and has comparable oncological results with open surgery. Lin et al [8] designed a randomized controlled trial to compare LRC and ORC in patients undergoing radical cystectomy for bladder cancer. They demonstrated that LRC was superior to ORC in perioperative outcomes, including EBL, blood transfusion rate, and analgesic requirement and there was no major difference in oncologic outcomes between LRC and ORC group. Tang et al. [15] and Aboumarzouk et al. [16] have also confirmed efficacy and safety of LRC by meta-analysis. With the continuous maturation of laparoscopic technology, robot-assisted radical cystectomy has become a treatment way for bladder cancer and its clinical feasibility has been recognized by more and more doctors [11]. Recently, Richards et al. [17] carried out a study to compare the safety and efficacy between robot-assisted radical cystectomy (RARC) and ORC in elderly patients ≥ 75 years old. The results revealed that RARC can achieve similar perioperative outcomes without compromising pathologic outcomes, with less blood loss and shorter hospital stays. Therefore, LRC or RARC for the elderly has a good minimally invasive effect and short-term effect. However, as far as we know, reports regarding to complications and survival analyzes of LRC or ORC in elderly patients over 75 years are rare.

In this study, LRC group required longer operative time and had less EBL compared with ORC group. What is more, LRC significantly reduced the incidence of ileus and perioperative infection within 90 days after surgery compared to ORC group. Hb and ALB, which reflect the body's nutritional status and strongly associated with 90-day mortality [18], both significantly decreased in LRC and ORC groups compared with preoperative data. Therefore, postoperative nutritional balance is particularly important for all patients in both groups, and can greatly reduce the incidence of delayed recovery. Overall survival and progression-free survival are recognized as the gold standard for evaluating the efficacy of tumor therapy. In this study, it was observed that LRC gained comparable overall survival (*P* = 0.726) and progress-free survival (*P* = 0.696) compared with ORC at a median follow-up of 28 months (range 12–48 months).

There are several points to note in laparoscopic surgery. First of all, the main organ function should be fully assessed before surgery, cardiac function, lung function, liver function and nutritional status should be fully adjusted, and blood sugar and blood pressure should be well controlled. Secondly, the surgeon should have a good experience of laparoscopic radical cystectomy. Under the premise of low abdominal pressure and ensuring the radical effect of cancer, surgeons should try to shorten the operation time to reduce the influence of CO2 on cardiopulmonary function. In addition, intraoperative hypothermia are closely related to postoperative complications, such as wound infection, coagulation or circulatory dysfunction, and even increase the mortality rate [19]. Therefore, the prevention of hypothermia appears to be particularly important. In our hospital, warm facilities are routinely used.

Because of small incision and mild pain in laparoscopic surgery, patients could be encouraged early ambulation and deeply breathing, which are conducive to the prevention of deep vein thrombosis and pulmonary complications.

This study has some limitations. First of all, the sample size of this study was small. In most cases, patients aged over 75 years old, especially those in poor physical condition, would refuse to undergo such a major surgery. Therefore, the results maybe only reflect the outcome of those with better physical condition. Secondly, this study is a single-center trial, and inevitably has selection bias. In the future, we will design a multicenter, randomized controlled trial and expand the sample size to achieve more reliable results.

In conclusion, this study confirmed that LRC could achieve similar tumor treatment efficacy compared to ORC, with fewer perioperative complications and less blood loss. Therefore, we suggest that LRC should be considered as the primary intervention for patients aged over 75 years old with muscle invasive bladder cancer or non-muscle invasive bladder cancer with high risk factors. However, larger sample size and multicenter prospective randomized controlled trial are needed to further confirm these results.

## MATERIALS AND METHODS

### Patients

**Figure 2:**
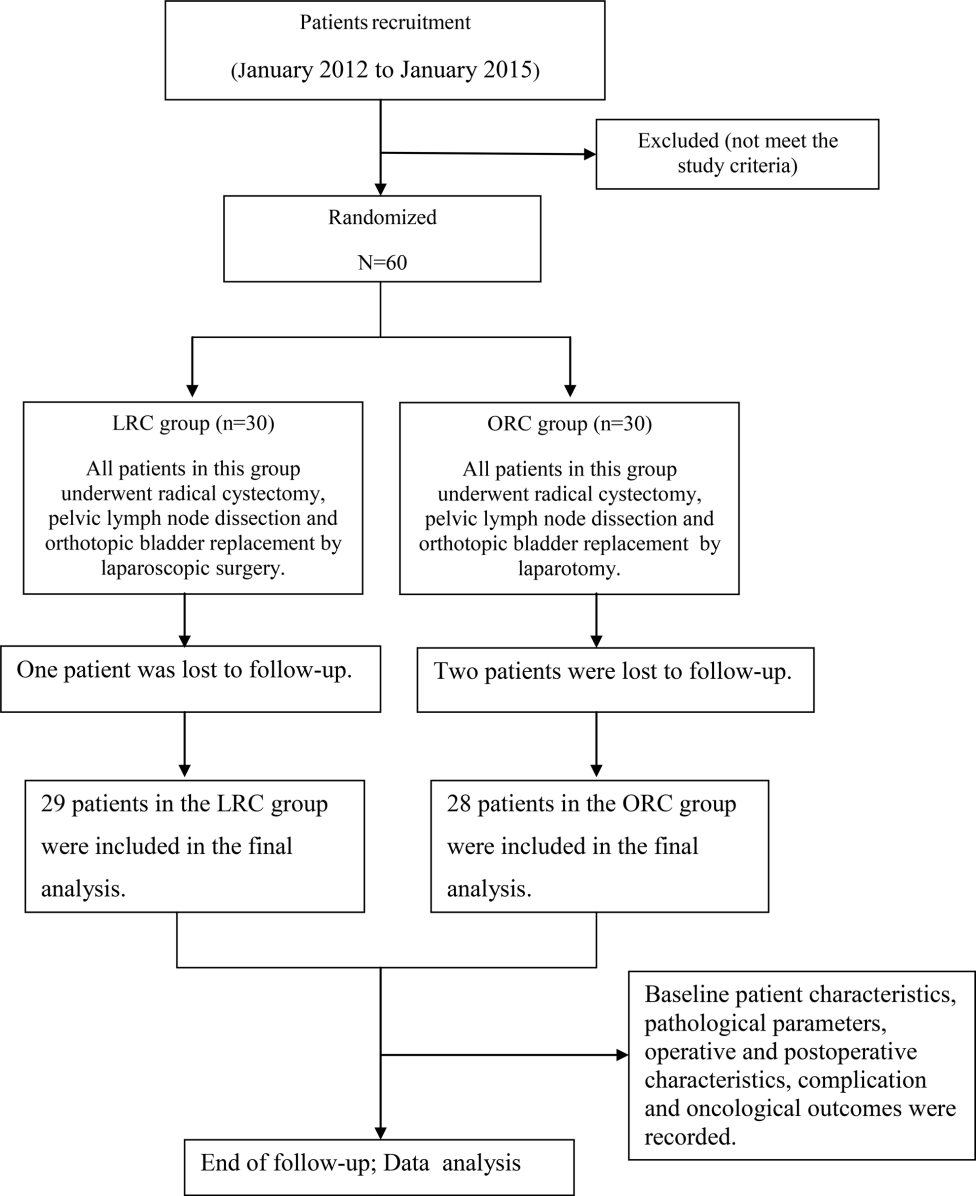
The trial flow chart

This study was approved by the Ethics Committee of The First Affiliated Hospital of Chongqing Medical University. All study participants provided written informed consent before participation. This clinical trial was a prospective open-label, randomly controlled trial to assess compare the morbidity, mortality and oncological results between LRC and ORC in the elderly patients over 75 years old. The overall flow chart of this study was shown in Figure 2. Patients included strictly follow the inclusion and exclusion criteria.

The inclusion criteria were as follows: 1) > 75 years old; 2) histologically diagnosed as muscle-invasive bladder cancer by transurethral resection, or biopsy confirmed recurrent multifocal high-grade superficial bladder cancer or bladder cancer *in situ* that were refractory to repeated transurethral resection; 3) a life expectancy of ≥ 5 years; 4) an Eastern Cooperative Oncology Group (ECOG) Performance Status of 0–2; 5) hematology, liver and kidney function are normal; 6) good understanding and compliance by patients with the pilot program, and provision of informed consent. The exclusion criteria were as follows: 1) patients with clinical or radiologic evidence of distant metastases; 2) a history of cancer, abdominal surgery and the recent acute infection; 3) tumor impregnated with serous or stage IV according to intraoperative assessment; 4) receiving preoperative radiotherapy or chemotherapy; 5) with contraindications of anesthesia or pneumoperitoneum; autoimmune diseases; 6) metabolic diseases or major diseases of other systems.

Between January 2012 and January 2015, 60 patients were recruited into this study. Patients would be randomly assigned in a 1:1 ratio to either LRC or ORC group according to the random number table. The treatment regimens for the two groups were as follows.

### Treatment

All patients underwent a preoperative systemic examination, including routine laboratory test, chest radiography, and intravenous pyelogram, echocardiography, lung function test, computerized tomography or magnetic resonance imaging and abdominal ultrasonography. Patients were graded according to the American Society of Anesthesiologists (ASA) class. Common comorbidities such as hypertension, coronary artery disease, chronic obstructive pulmonary disease, diabetes mellitus and other chronic diseases were recorded.

All patients started a semi-liquid diet two days before surgery and a liquid diet one day before surgery. All patients underwent intestinal preparation with 50% magnesium sulphate at 12 hours prior to surgery. Patients wore elastic stockings before entering the operating room and began intravenous injection of broad-spectrum antibiotics during anesthesia induction. All the operations and perioperative management were performed by the same experienced surgical team. Procedure of bilateral lymph node dissection for male and female patients in LRC and ORC groups was performed according to Campbell-Walsh Urology [20]. Patients were taken head low-foot high supine position on the basis of satisfactory general anesthesia.

ORC was performed as follows. After a median incision of lower abdomen with length of 15–20 cm, total removal of bladder and prostate and pelvic lymph node dissection by retroperitoneal approach were conducted. 10–15 cm ileum was intercepted to serve as an outflow tract. Ureter was trimmed to a ramp and inserted into the ileal outflow tract for anastomosis. Indwelling ureteral stent was led from the distal ileum. The ileum outflow tract was output and fixed at a oval incision at the right lower quadrant of abdomen. If necessary, hysterectomy and ovary removal were carried out for female patients.

LRC was carried out as follows. Using 5-point puncture, abdominal pressure maintained at 10–15 mm Hg. Pelvic lymph node dissection and radical resection of bladder was performed under laparoscope. The specimen into the specimen bag was taken out completely by a 4–5 cm incision at the lower abdominal medialline. The lower ureter was pulled out of the incision and ureteral stent was placed into the ureter. The procedure of ileus outflow tract formation and ureteral planting was the same as the ORC group.

### Follow-up

Upon completion of treatment, patients were re-evaluated every month for the first three months, every 3–6 months thereafter. Check items include renal function, abdominal B-ultrasound, chest X-ray and pelvic CT.

### Parameters and endpoint

The baseline patient characteristics included age, gender, body mass index (BMI), comorbid conditions, surgery history, laboratory test results, and ASA class. Pathological parameters included pathological stage, grade, lymph node status and number, and positive surgical margins. Pathological tumor stage and grade were evaluated according to the TNM classification in NNCN guidelines of 2012. Operative characteristics contained operative time, estimated blood loss (EBL) and transfusion needed, while postoperative characteristics included hemoglobin (Hb), serum creatinine(SCR), serum albumin (ALB), time to liquid intake, time to nasogastric tube removal, time to canalization, time to exsufflation, and hospital stay after surgery. Complication that occurred within 90 days after operation were considered as acute complications, including infection, ileus, anastomotic leak, delirium, arrhythmia and wound dehiscence. Those that occurred 90 days or later after operation were defined as late complications, including pyelonephritis, ileus, ureteral stricture and incisional hernia. Oncological outcomes, including survival and recurrence were evaluated.

### Statistical analysis

Statistical Package for Social Scientists (SPSS, version 19.0, IL) was used for all analyses. Measurement data was showed by $\bar{X}\pm S$ and comparison between the two groups was conducted by *t*-test for two independent samples. The data before and after surgery in the same group were compared by *t*-test of paired data. Count data was showed by the frequency (%), and comparison between the two groups was completed by χ^2^ test or Fisher exact probability method. Kaplan-Meier method was used for survival analysis. The log-rank method was used for the comparison of nonparametric survival analyzes between the two groups. *P* < 0.05 was considered to be statistically significant.
